# Now for something completely different: *Prototheca*, pathogenic algae

**DOI:** 10.1371/journal.ppat.1009362

**Published:** 2021-04-01

**Authors:** Christopher D. Shave, Linda Millyard, Robin C. May

**Affiliations:** Institute of Microbiology & Infection and School of Biosciences, University of Birmingham, Birmingham, United Kingdom; McGill University, CANADA

## What are *Prototheca*?

**Fig 1:**
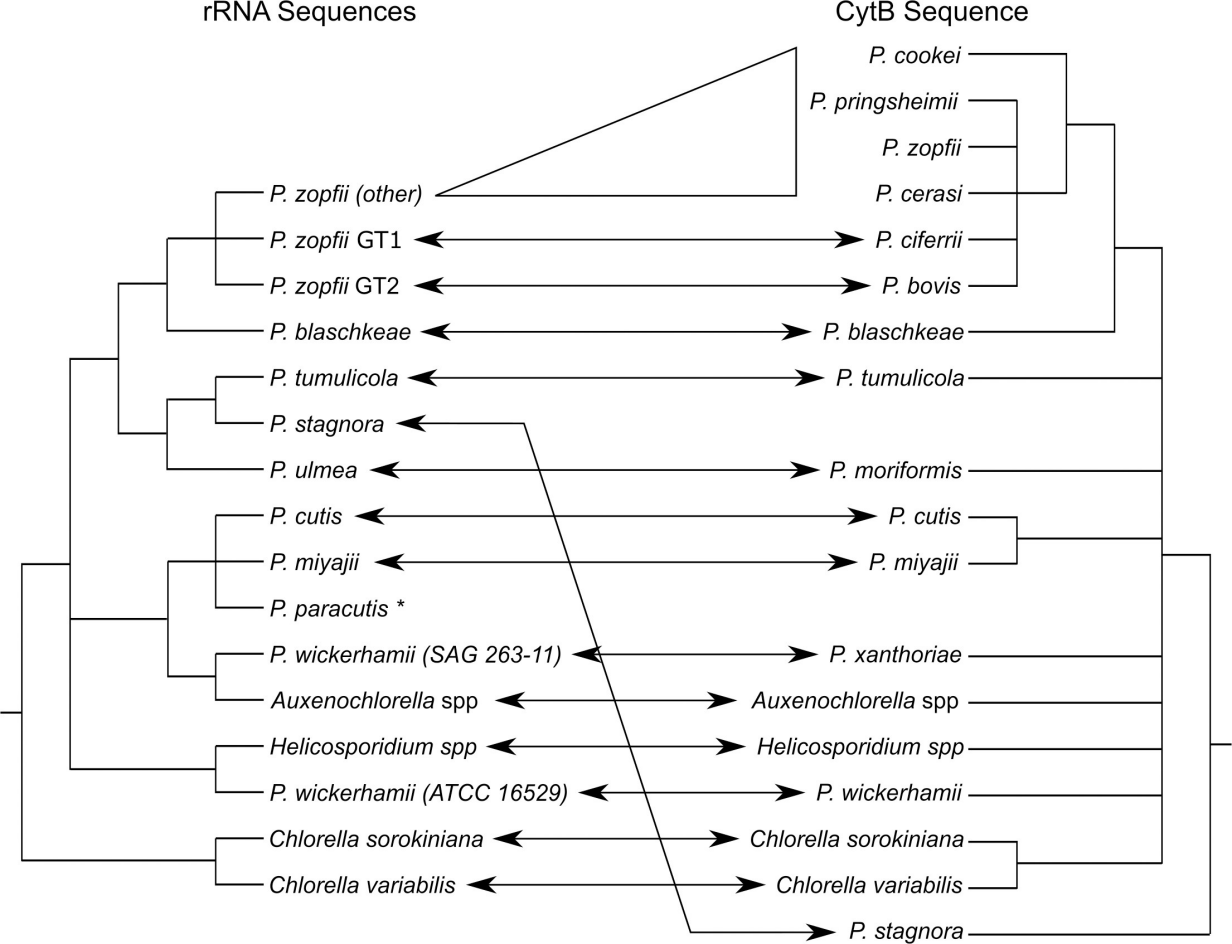
Probable relationships between species/genotypes within the *Prototheca/Helicosporidium/Auxenochlorella/Chlorella* lineage. Left—a consensus cladogram built from analyses using predominantly ribosomal RNA (small subunit, internal transcribed spacer region, and D1/D2 region of the large subunit) sequence data [2–5]. Species names are defined by a mixture of assimilation profiles, growth conditions, and sequence data. Right–a cladogram built using a partial mitochondrial cytochrome b sequence [1]. Species names are defined by clustering and cytochrome b sequence similarity. Arrows between the trees indicate equivalent species, where renaming has occurred. All nodes shown are supported by bootstrap values greater than 70 in at least one analysis. Branch lengths are arbitrary. *Discovered recently and therefore not included in the cytochrome b analysis.

Members of the genus *Prototheca* are nonphotosynthetic algae, closely related to the well-known green algal genus *Chlorella*. This “genus” encompasses the additional genera *Auxenochlorella* and *Helicosporidium* (Fig 1). Together, this collection of genera is referred to as the AHP lineage, though relationships within the lineage remain unclear [1–5]. Analyses based on the mitochondrial cytochrome b sequence suggest this lineage may include *Chlorella* species (Fig 1), which might accordingly be called the CHAP lineage [1–5].

## Why are we talking about an alga in *PLOS Pathogens*?

Lacking chlorophyll, *Prototheca* species are obligate heterotrophs, and six are opportunistic pathogens of vertebrates. Of particular interest are the former species *Prototheca zopfii* (specifically the lineage known as genotype 2, recently raised to species status as *Prototheca bovis* [1]) and the current species *Prototheca wickerhamii*, which are the main causative agents for cattle and human infections respectively [6,7]. *P*. *bovis* and *P*. *wickerhamii* also maintain the largest host ranges, including: cats, dogs, buffaloes, horses (*P*. *bovis* only), and goats (*P*. *wickerhamii* only).

Other pathogenic algae exist, though *Prototheca* are the most significant in terms of the number of infections and their predilection towards humans and domesticated animals. Members of the nonphotosynthetic genus *Helicosporidium* are pathogens of invertebrates, particularly insects [8]. Photosynthetic algae (*Chlorella* and *Desmodesmus*) have also been reported to infect mammals [9,10].

## How do *Prototheca* infections occur?

Infections in cattle typically present as mastitis (inflammation of the mammary tissue). Infections are usually subclinical—detectable only through raised somatic cell counts and the presence of *Prototheca* in milk—but clinical mastitis has both acute and chronic presentations, both of which reduce milk yield [6,11]. Acute mastitis is associated with raised temperature, pain, and swelling, while chronic mastitis is associated with permanent damage to alveoli and mammary parenchyma. Infections are typically restricted to the mammary tissue, but rare cases of systemic infection have been reported [12].

**Fig 2:**
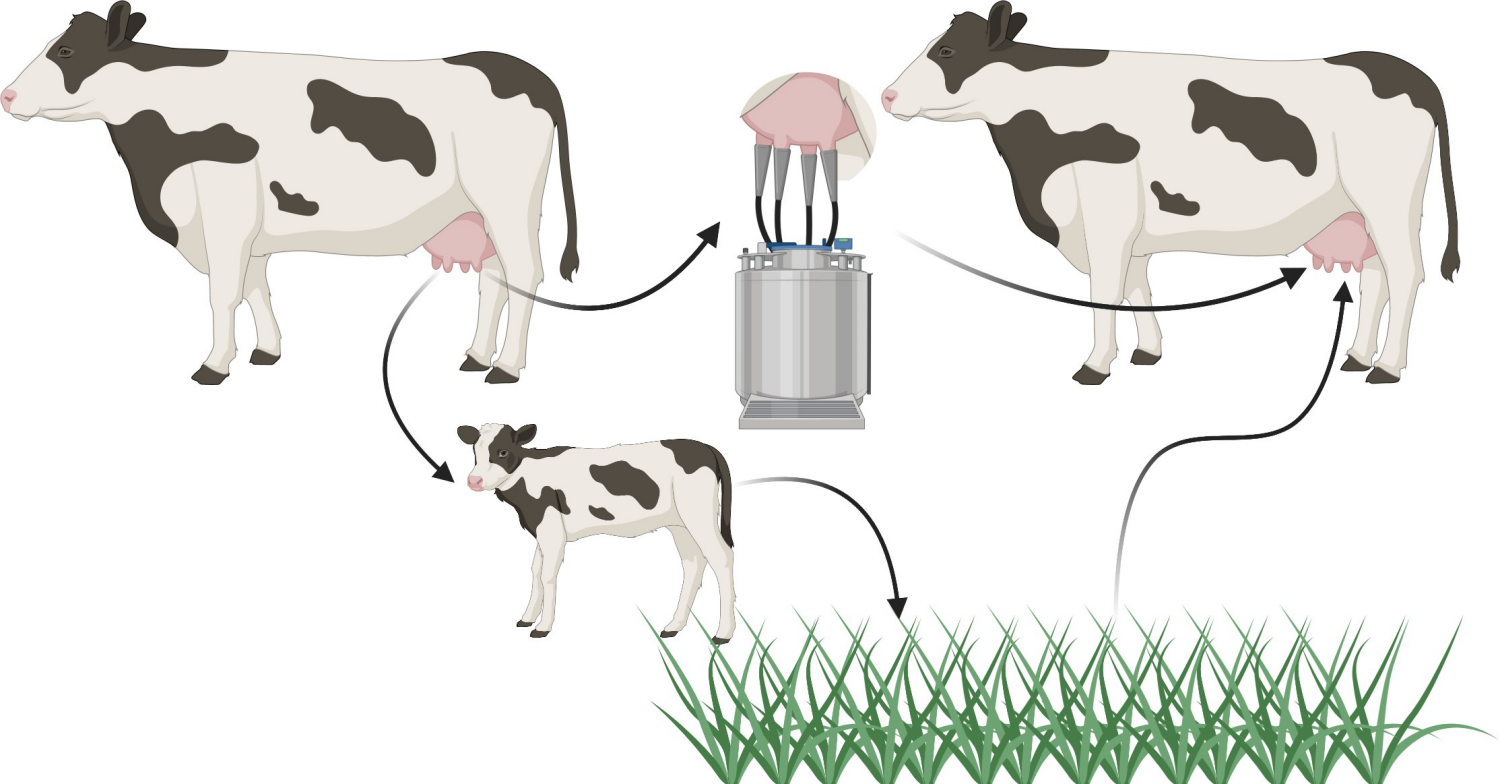
Possible infection cycle for *Prototheca bovis*. An udder infection results in the presence of *Prototheca* cells in milk. These cells may be ingested by calves and excreted into the environment. Contaminated milk may also result in *Prototheca* cells being present on milking machinery, representing a more direct method of transmission. Reentry into an udder is likely through the teat orifice contacting contaminated surfaces. Created with BioRender.com.

*Prototheca* appears responsible for a nonnegligible proportion of bovine mastitis cases (1.2% to 13.3% of Polish samples, 11.2% of Italian herds affected [6,13,14]), though the transmission cycle is currently speculative (Fig 2). Entry is probably through the teat orifice, from contaminated milking equipment or environmental sources [11]. *Prototheca* cells in milk may return to the environment in the faeces of calves [15]. There is currently no economically viable treatment, and spontaneous recovery is rare to nonexistent [11]. Consequently, affected cows are culled, representing a large economic and animal welfare burden.

Presentation and prognosis of human infections are highly dependent on the site of infection, which is in turn strongly influenced by the host’s immune status. The majority of infections are restricted to localised skin lesions, but joint infection (particularly the elbow) and disseminated infection (affecting a range of organs, each with unique presentations) are more prevalent in immunocompetent and immunocompromised individuals, respectively [16]. Almost all human infections become chronic, with death usually associated with disseminated infections [17]. Human infections probably occur through environmental contamination of a wound, with little evidence for direct human-to-human transmission [16]. Reports of infection from contaminated dairy products exist but are infrequent. Given the differences between human and cattle *Prototheca* pathogens, contaminated dairy is unlikely to pose a public health risk.

Human and cattle infections have been reported from all permanently settled continents except, currently, in cattle in Africa; likely reflecting diagnostic challenges rather than a true absence of the pathogen [6,16]. Historically, infections by *Prototheca* species have been diagnosed through a combination of histopathology, morphology, and culturing methods, with biochemical assimilation assays providing species-level resolution. However, protothecal infections have been misdiagnosed (usually as fungal infections) or missed (in subclinical cases) obscuring the true prevalence and distribution of infections. More widespread use of molecular techniques, including PCR-restriction fragment length polymorphism (RFLP) assays and matrix-assisted laser desorption/ionization time-of-flight (MALDI-ToF) mass spectrometry, will hopefully improve this situation [3,18].

Unfortunately, few environmental studies have taken place for *Prototheca*. Those that have almost always investigate the surroundings of potential hosts. Our understanding of the natural ecology of *Prototheca* species is therefore lacking. *P*. *wickerhamii* is the most abundant species in human sewage [19], and species formerly identified as *P*. *zopfii* (either *P*. *bovis* or *Prototheca ciferrii* [1]) tend to be the most abundant around cattle [6], but the abundance of any species away from their preferred host remains unknown. In environments where *Prototheca* have been identified, they are present in a variety of contexts including: tree slime flux; rivers and ponds; mud; faeces/sewage (human, cattle, pigs, dogs); food (human and cattle); and industrial waste [16,19]. *Prototheca* have been found to colonise animals nonpathogenically and transiently [19,20].

## How are algae capable of causing disease?

The mechanisms by which algae are able to infect hosts and cause disease are currently unknown. Adaptations that facilitate *Prototheca* pathology in particular are also unknown. Genomic and proteomic approaches to identify virulence factors have been stymied by a lack of supporting information. Typically, only single genomes per species are being published, further complicating identification of relevant virulence determinants from individual variation.

Protothecal infections typically develop over months, indicating *Prototheca*’s ability to survive or evade host immunity is a key component of their pathology. To this end, *Prototheca* cells are able to survive digestion by macrophages and appear to replicate within the phagolysosome [21]. Furthermore, *P*. *bovis* has been shown to kill phagocytic cells even after phagocytosis was blocked [22]. Killing was restricted to phagocytic host cells, and the former *P*. *zopfii* genotype 1 (raised to *P*. *ciferrii* [1]) could not kill these cells, suggesting that specific host-directed toxin(s) may be important for the virulence of *P*. *bovis*.

One possibility is that, rather than bespoke virulence factors, *Prototheca* may exploit environmental adaptations for “accidental virulence,” as has been proposed for other eukaryotic pathogens [23]. The presence of closely related endosymbiotic algal species may suggest one such exaptation. The genera *Chlorella* and *Auxenochlorella* both contain species that are known endosymbionts of organisms such as the ciliate protist *Paramecium bursaria* and the cnidarian *Hydra viridis* [24–26].

Current models require *Chlorella* to survive digestion by their prospective host to establish endosymbioses [24]. Additionally, *Chlorella* endosymbionts exist in a wide range of host protists and invertebrates [24,27]. This may indicate that processes that enable endosymbionts to survive digestion in one organism may be generalisable to other hosts. If mechanisms to survive digestion facilitate either endosymbiosis or parasitism, we may expect some pathogens to be closely related to endosymbionts—as occurs within the AHP/CHAP algal lineage. *Desmodesmus*, an unrelated pathogenic alga, also has close endosymbiotic relatives [9,28].

Another feature potentially predisposing *Prototheca* to pathology is the ability to form biofilms in isolation [29,30], while biofilm formation in *Chlorella* is limited without a microbial community [31]. Biofilms have been proposed to play important roles in immune evasion and drug resistance of many pathogens, and biofilm formation appears to correlate with pathogenicity in *Prototheca* species [29,30,32]. Biofilms appear to increase the resistance of species formerly identified as *P*. *zopfii* (likely *P*. *bovis*) against various sanitizers, potentially enhancing transmission by preventing removal from contaminated surfaces [33]. Peripheral blood mononuclear cells produce IL-6, an early pro-inflammatory cytokine, in response to planktonic *P*. *wickerhamii* but not *P*. *wickerhamii* biofilms, thus potentially enhancing immune evasion [29].

## What do we still not know?

Quite a lot. The fundamental differences between pathogenic and environmental species, if any, remain unknown. We do not know if *P*. *bovis* and *P*. *wickerhamii*, which are relatively distantly related species within the lineage (Fig 1), use similar mechanisms for pathogenesis.

*Prototheca* host preferences are poorly understood. For example, *P*. *wickerhamii* and *P*. *bovis* dominate human and cattle infections, respectively, but seem equally prevalent in buffaloes [34]. The reason for the uniquely aggressive progression of *Prototheca* infection in dogs, which is usually fatal, is also unknown [35].

From an evolutionary perspective, it is unclear whether *Prototheca* benefit from pathology. Dispersal in milk and faeces is a potential advantage for *P*. *bovis* from infecting cattle (Fig 2), but there is no obvious mechanism of egress for *P*. *wickerhamii* from humans.

What drives success or failure of an immune response against *Prototheca*, in any host, is unknown. Neutropenic cancer patients and transplant recipients are at particular risk, potentially highlighting the importance of neutrophils. By contrast, those with severely depleted CD4+ cell counts (as a result of HIV infection) are not as severely affected as one might expect, suggesting that T-cell responses may be less important [36].

Finally, our understanding of how *Prototheca* infections respond to treatment is insufficient. As the only pathogenic algae of note, treatment usually involves surgical removal and/or antifungal drugs with mixed efficacy. Unfortunately, *in vitro* susceptibility tests are poor predictors for success of existing antiprotothecal treatment [37]. There have been notable cases of treatment failure when isolates seemed susceptible or success when isolates seemed resistant, as well as unpredictable changes in susceptibility during the course of treatment [38,39]. Recent *in vitro* work has revealed promising, novel algicidal treatments, but their *in vivo* efficacy remains to be seen [37,40,41].

## Conclusions

*Prototheca* and their relatives represent a fascinating but poorly understood class of pathogens. A deeper understanding of their genomes and cell biology holds great potential, both in terms of improving the treatment of animal and human infections and in shedding light on principles that underlie pathogenesis in general.
